# Mind the gap: Coping with delay in environmental governance

**DOI:** 10.1007/s13280-019-01265-z

**Published:** 2019-09-30

**Authors:** Mikael Karlsson, Michael Gilek

**Affiliations:** 1 Division of Philosophy, KTH Royal Institute of Technology, 100 44, Stockholm, Sweden; 2 School of Natural Sciences, Technology and Environmental Studies, Södertörn University, Alfred Nobels Allé 7, 141 89 Huddinge, Sweden

**Keywords:** Decision-making, Delay, Environmental goals, Environmental science and policy, Science denial

## Abstract

Gaps between public policy goals and the state of the environment are often significant. However, while goal failures in environmental governance are studied in a number of disciplines, the knowledge on the various causes behind delayed goal achievement is still incomplete. In this article we propose a new framework for analysis of delay mechanisms in science and policy, with the intention to provide a complementary lens for describing, analysing and counteracting delay in environmental governance. The framework is based on case-study findings from recent research focusing on goal-failures in policies for climate change, hazardous chemicals, biodiversity loss and eutrophication. It is also related to previous research on science and policy processes and their interactions. We exemplify the framework with two delay mechanisms that we consider particularly important to highlight—denial of science and decision thresholds. We call for further research in the field, for development of the framework, and not least for increased attention to delay mechanisms in environmental policy review and development on national as well as international levels.

## Introduction

Environmental governance entails both successful cases and fundamental failures. While some problems have been adequately managed, the lack of goal achievement is generally striking. The positive cases include mitigation of local pollution, for example decreased nutrient load into many freshwater systems (Le Moal et al. 2019), and regional recovery of some endangered species, e.g. the white-tailed eagle in the Baltic Region (HELCOM 2016), but also global accomplishments, such as decreased impact on the ozone layer (WMO 2018). These and other examples show that policies and voluntary measures may give positive results. Despite this, it is far more common that environmental goals are not achieved, on global as well as regional and national levels. The gaps between environmental goals and the state of the environment are often significant.

On the global level, overall goals for avoiding dangerous climate change under the UNFCCC (UNEP 2018), for conserving biodiversity under the CBD (CBD 2014) and for minimising adverse effects of chemicals, as set out at the World Summit for Sustainable Development (UNEP 2019), are far from met. In the EU, the key objectives in the 7th Environmental Action Programme are out of reach (EEA 2018). On national levels, environmental goals are similarly often not achieved (OECD 2001), for example in the environmental forerunner country Sweden, where only 1 out of 16 national environmental quality objectives set by the parliament is assessed to be reachable by the set target date (SEPA 2019). Considering trends and interim targets, the picture is often more complex, with plenty of examples of backsliding, but also of progress, not seldom in the short term, meaning that overall goals may be reached in the future, but later than what has been agreed in the democratic political process (EEA 2018; SEPA 2019). Progress is actually taking place in many cases, albeit with delay. Paradoxically, delay is sometimes occurring even when the body of knowledge on problems and solutions is solid, when goals are democratically balanced and agreed, and when mitigating strategies are technologically available and economically feasible or even profitable. This latter situation is observed, for example, in climate policy, where societal co-benefits of instruments and measures are estimated to be significantly larger that mitigation costs (Karlsson and Westling 2017). However, while much research in various disciplines has been devoted to science and policy processes in relation to environmental goals, the knowledge on various causes for delay is still incomplete.

This perspective article explores the mechanisms behind delay in science and public policy, with the aim to improve the understanding of goal-state gaps in environmental governance, and how these can be better analysed and bridged. The intention is to complement existing research and theories by introducing a framework that can assist in the assessment of goal achievement by highlighting specific critical issues and processes that can cause delay. In contrast to several existing approaches that analyse policy and implementation gaps, the proposed framework spans over both the science and policy domains. Here, we share the understanding of Jenkins-Smith et al. (2017) that the “purpose of a framework is to provide a shared research platform that enables analysts to work together in describing, explaining, and, sometimes, predicting phenomena within and across different contexts”. The article calls for paying more attention to delay mechanisms in research, the generation of science-based advice and environmental governance practice. However, further studies on delay mechanisms are indeed needed to develop, refine and operationalise the presented framework, its assumptions, concepts and their relations.

The next section describes some present approaches with relevance for delay in environmental governance, which leads over to an elaboration of a new framework. This is followed by a section in which we exemplify aspects of the framework through two delay mechanisms, which we in a recent research projects have found important—denial of science and decision thresholds. The article ends with a discussion.

## Environmental governance and delay

Gaps between environmental goals and the state of the environment are not unstudied in environmental research. On the contrary, lack of goal achievement in public policy is researched since long in several disciplines. In natural and technical sciences, focus is placed on advancing the understanding of the problem at hand, thereby reducing uncertainty for decision-makers, and on engineering solutions of various kind. Environmental sociologists focus on e.g., societal change and how to characterise environmental risk, including for example, scholars who assert explanatory power to theories on Ecological or Reflexive Modernisation, sometimes constituting a basis for normative prescriptions (Karlsson 2005; Boström et al. 2017; Machin 2019). In environmental psychology, issues relating to, for example, behaviour and change are explored (Steg and Vlek 2009; Gifford and Nilsson 2014). Environmental economics and law rather study how to manage market failures (Baranzini et al. 2017; Shmelev 2017) and shortcomings in legal systems (Jóhannsdóttir 2009; Rakhyun and Bosselmann 2013).

Science and technology studies as well as environmental risk governance research give attention to interactions between science and natural systems on the one hand, and policy and social systems on the other (van Asselt and Renn 2011; Linke et al. 2013; Gilek et al. 2016). Studies on risk decision-making and environmental goal-setting are found in, for instance, regulatory toxicology and environmental philosophy (Sandin et al. 2002; Karlsson 2006; Edvardsson 2007). Well-developed theorising of relevance for this Perspectives article is carried out not least in the domain of political sciences. The field of policy studies includes the substance-focused branch of policy analysis (knowledge in policy) (Walker 2000; Runhaar et al. 2006), as well as policy process research (knowledge of policy) (Weible 2017). Within the latter, a number of theories are concerned with policy development, change and implementation, for example the Advocacy Coalition Framework (ACF), the Institutional Analysis and Development framework, the Multiple Streams Framework (MSF), and the Innovation and Diffusion Models (Ruseva et al. 2019). Sometimes, different approaches are applied in parallel to describe a complex environmental policy situation (Eriksson et al. 2010).

In the referred literature, an array of different interconnected, overlapping and sometimes unrelated theories, frameworks, models and concepts is used to describe and explain reasons for gaps, and barriers against bridging them. Common concepts include science denial (Edvardsson et al. 2017), scientific uncertainty and disagreement (Karlsson 2005; Saunders et al. 2017), non-rational goals (Edvardsson 2007), politicisation (Eriksson et al. 2010), institutional lock-in (Unruh 2000), governance barriers (Tynkkynen 2017), burden of proof requirements (Sandin et al. 2002; Karlsson 2005; Alfredsson and Karlsson 2016; van den Bergh 2017), mismatch (Gilek and Karlsson 2016), and implementation constraints (Hassler 2017). Broader explanations are given by not least the ACF, which centres on policy subsystems and actors (Jenkins-Smith et al. 2017). In the ACF, people operating within an advocacy coalition are considered to share common belief systems, including a stable deep normative core, a set of specific policy core beliefs, and related, and more changeable, secondary beliefs or aspects. In pursuing their respective interests within policy subsystems, coalitions negotiate and learn gradually from e.g., policy implementation results, but can also be influenced by internal or comparatively dramatic external events. These factors interact and can potentially lead to policy change, albeit after long time in the normal case. The MSF is also of relevance for delay, not least the insight that even when the three problem, policy and political streams couple, agenda change is often dependent on various windows of opportunity, which policy entrepreneurs may not always be able to influence (Herweg et al. 2017).

Obviously, there are advantages and drawbacks associated with all of the referred concepts, frameworks and theories, which all help in analysing the degree of goal achievement in environmental policy. For example, it can be argued that the ‘barrier’ concept, while pointing out key challenges to work with to promote environmental goals, can also give the impression of a total common standstill from one policy stage to another, e.g., between policy formulation and implementation. While such gridlocks are not unheard of in environmental governance, the more usual situation, for example, in marine environmental governance in the Baltic Sea (Karlsson and Gilek 2018), is rather that of delayed progress. Other concepts, like ‘scientific uncertainty’ or ‘non-operational goals’, can more be seen as basic challenges or problems in science and policy, respectively. Our aim is rather to present a framework that can be used as an analytical lens in research and not least policy practice, to target mechanisms in specific cases of delay, and to potentially compare these. While the ACF, MSF and other policy process theories and frameworks have much to offer, they direct the focus mainly to the policy sphere and processes on a rather generic and long-term level. In many situations, we would argue that the actual mechanisms of delay are linked to more specific aspects in both science and policy. However, to what extent the approaches described have explanatory value is often contextual. Considering this, our intention is neither to pen down when a specific approach is applicable or not, nor to review and synthesise this broad literature and upon that present new theory. Instead, we want to highlight the existence of delay mechanisms as a complement to other approaches, and in doing so also help to analyse them in practice to ultimately improve the understanding of how delay can be counteracted.

A number of empirical studies show that delay may occur over the entire science-policy field, which should be kept in mind in any analysis. An early example is the study by Thelander and Lundgren (1989), who identify several delay mechanisms linked to the scientific discovery, communication and politics of environmental problems, for example concerning various hazardous chemicals. Nolin (1995) similarly describes the more than a decade long process from scientific discovery to political measures concerning ozone depleting substances. Two comprehensive reports from the European Environment Agency (EEA 2001, 2013), moreover, investigate late policy lessons from early scientific warnings, in a number of case studies—on, for example, various hazardous chemicals, climate change and ozone depletion—where delay, for various reasons, has been significant. While these previous studies indeed are informative, we believe that even more knowledge could have been generated if the findings would have been placed into a general framework that allows systematic and comparative analysis.

**Fig. 1:**
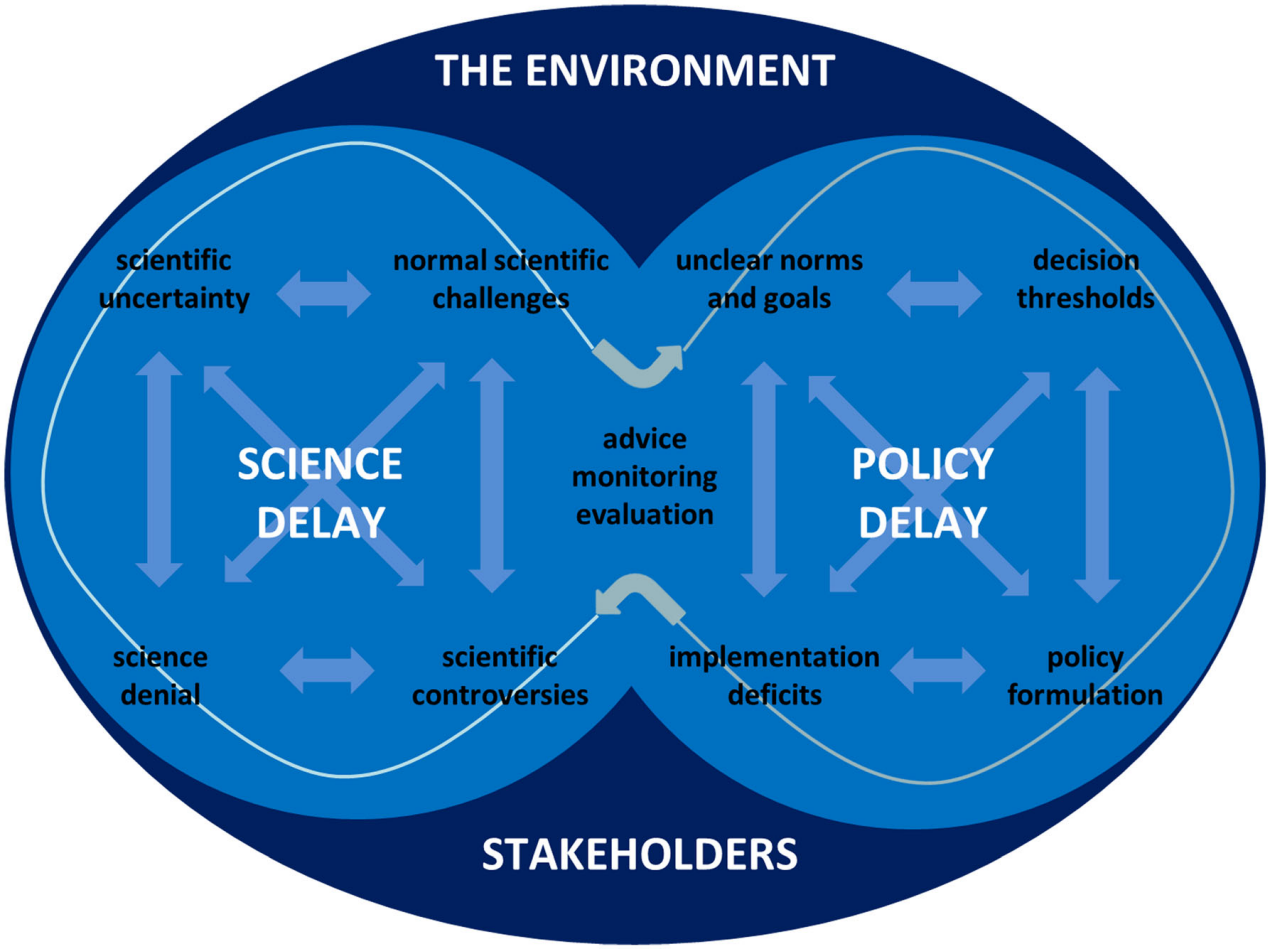
A framework for analysis of delay mechanisms in environmental governance

To concretise this, we will next propose a new framework over central delay mechanisms that ought to be further analysed and compared in environmental governance research and practice (Fig. 1). Thereafter, we exemplify the framework with two, out of a wider set of, delay mechanisms from the science and policy domain, respectively—denial of science and decision thresholds. While we, based on previous research, consider these mechanisms to be of central interest, we do not imply that other potential delay sources highlighted in Fig. 1 necessarily are less significant.

### Developing a framework for analysing delay in environmental governance

The presented framework is based on the recognition that (i) delay can be caused by several different mechanisms in both the science and the policy domain; (ii) multidirectional interactions between and within the science and policy spheres have fundamental importance for both the emergence and mitigation of delay, and (iii) a multitude of delay mechanisms can be at play simultaneously on several levels, and that the playing field may differ substantially from one case to another. This means that, while we agree with e.g. Varjopuro et al. (2014), who explicitly explores delay linked to eutrophication in the Baltic Sea, that delay may be manifested at different places, we argue that contrary to their rather linear separation of decision-making followed by implementation delay, it is important to acknowledge the multiple sources of delay, as well as the interactions among these. We, moreover, recognise the fundamental differences between the system-to-be-governed and the governing system (Jentoft and Chuenpagdee 2015), where delay in the former in terms of natural processes (e.g., internal nutrient dynamics, environmental persistence of hazardous chemicals, etc.) is seldom governable per se, in contrast to the latter, where governance decisions are made (e.g. on measures to mitigate total nutrient loads as well as chemical pollution). Identifying inherent ecosystem mechanisms that can delay recovery from e.g. eutrophication and chemical pollution (e.g. nutrient release from sediments, and persistence of substances, respectively) is obviously important when elaborating adequate governance strategies. Such strategies, however, still need to be developed outside the ecosystems, in the governing system where delay has another character.

The framework we propose is built around potential delay mechanisms in the two spheres of science and policy (Fig. 1). It is important to note that our aim is to draw attention in environmental governance research and practice to these mechanisms and we, therefore, rather pragmatically focus on a set of issues of importance for goal achievement, based on previous empirical studies in the field. Obviously, also other mechanisms may contribute to delay, and we hope to stimulate further studies in that respect, as well as of potential underlying factors. However, the subsequent section presents some illustrative examples relating to climate, chemicals, biodiversity and marine environmental policy, being four areas where severe problems persist despite democratically agreed and ambitious policies.

First, in relation to the science domain in Fig. 1, delay can occur in the production, interpretation and appraisal of knowledge, as well as in the generation of science-based advice in relation to environmental goals, and in scientific monitoring and the evaluation of policies. Here, practical challenges are linked to, for example, generation, understanding and evaluation of data, knowledge, and scientific uncertainty, various theory-based and methodological controversies between different scientific approaches, lack of sufficient consensus-promoting arrangements, as well as lack of sufficient resources and incentives for policy-relevant research. The interpretation and use of scientific knowledge and science-based advice may be quite challenging in the policy context, if scientists strongly disagree or are unable to reach consensus (Haas 1992, 2004; Saunders et al. 2017), and is particularly problematic if various stakeholders deny science and employ science-denying strategies (Edvardsson et al. 2017). This means that delay can occur due to science denial, scientific controversies, scientific uncertainty, and normal scientific challenges, as illustrated in Fig. 1. Compared to Varjopuro et al. (2014) we consequently broaden what they label as the monitoring and evaluation step in delay, to the entire scientific field. Our assertion that delay can occur also in the two-way interactions between science and policy similarly broadens the picture and it underlines that processes may be far from rational and sequentially ordered. In the science to policy direction, it is clear that processes of building consensus within epistemic communities may increase the influence of science-based advice and thereby reduce delay (Haas 1992, 2004). To exemplify interactions in the other policy to science direction, it is often observed that politicisation of the science domain, e.g., in terms of how scientific panels or guidelines for risk assessments are set up, can lead to e.g., scientific controversies and loss of trust in science (Eriksson et al. 2010).

Second, within the policy domain, we contend that delay can be caused by a number of different mechanisms, at different places. As referred to above, policy processes can be described by a number of theories, frameworks and models. Evidently, these have developed over the years, from e.g., the policy cycle model to the ACF, and from the garbage can model to the MSF, and there is still a need for advancing the research in the field (Weible 2017). While the policy cycle model, with its criticised assumed ordered and rational links between different policy stages, can still allow for some analysis, the flow diagram of the ACF says more about the nature of policy change and explains why it often takes quite a long time to achieve in contested cases (Jenkins-Smith et al. 2017). The MSF similarly describes how agenda change and decision-making commonly presumes that a number of independent circumstances and factors coincide in practice. In addition to these and other common policy process research approaches (Ruseva et al. 2019), our new framework focuses more directly on a set of potentially important delay mechanisms and on their interactions. In Fig. 1, these include unclear norms and goals, decision thresholds, policy formulation challenges, and implementation deficits, the latter being a comparatively common research area. There are also important linkages to explore; if the operationalisation of goals, for example, does not meet adequate rationality criteria (Edvardsson 2007), the formulation of policy instruments may suffer, as will probably the subsequent implementation of these. Another delay mechanism highlighted in the framework in Fig. 1, being of particular importance and in need of additional attention in both governance research and practice, is decision thresholds, for instance various burden of proof requirements that are theoretically impossible or practically difficult to meet (Sandin et al. 2002; Karlsson 2005; Alfredsson and Karlsson 2016), and other types of obstacles (e.g. Keskitalo and Pettersson 2012).

In the following, we exemplify the new framework with one central delay mechanism per sphere in Fig. 1—denial of science and decision thresholds, respectively—to give some detail on issues in need of more attention, while still keeping the text concise. Even though some previous studies have focused on these two mechanisms they have not, to our knowledge, been placed within a broader framework for analysis of delay in environmental governance. In addition, previous research on environmental science denial has primarily centred on climate policy (Edvardsson et al. 2017), whereas much research on decision-making thresholds in environmental governance has focused on, for example, chemicals policy and regulation (Karlsson 2005). Against the background of a recent research project, we highlight a set of different cases selected to illustrate different situations in which delay occurs due to several factors, namely the four wicked (Alford and Head 2017) problems of climate change, chemical pollution, eutrophication and biodiversity loss, where goals have been shown to be difficult to reach (EEA 2018; SEPA 2019).

## Exemplifying delay through environmental science denial and decision thresholds

### Science denial

Despite strong scientific knowledge on negative human impact on the environment, much research shows that denial of science is far from uncommon (Edvardsson et al. 2017). Often the term ‘science denial’ is used for ‘an activity aimed at renouncing some well-justified assertion or theory in mainstream science’ (Hansson 2018). This should not be confused with scepticism, which is an important and natural part of the scientific process. The characteristics of science denial include conspiracy theories, fake experts, cherry-picked facts, extreme expectations on research, and discredit of scientists (Edvardsson et al. 2017), all being far from components of the scientific process. The most occurring and well-studied case is climate science denial, which can be divided into trend, attribution and impact denial (Rahmstorf 2004), or literal, interpretative and implicatory denial (Cohen 2001). While the former typology focuses on denial of natural scientific findings, the latter also concerns denial of the implications of knowledge, given democratically agreed goals. Numerous studies show that science denial impedes greenhouse gas emission mitigation, in particular in the U.S., and implicatory denial is geographically widespread.

Science denial occurs also in relation to other environmental problems, albeit less well-researched.^1^ One example is hazardous substances, where some stakeholders manufacture doubt on harmful effects of endocrine-disrupting substances (Bergman et al. 2015). This resembles the ozone layer depletion story, and many other examples of chemicals denial (EEA 2001, 2013; Karlsson 2019).^2^ Furthermore, in the area of wildlife conservation, researchers have claimed misuse of scientific results in different cases spanning from wolves in Sweden (Chapron 2014), over lions in Africa (Lindsey et al. 2013) and protection of boreal caribou in Canada (Boan et al. 2018), to exploitation of tropical peatlands (Wijedasa et al. 2017). In marine policy, a telling example of denial links to the role of phosphorus (and phosphates in detergents) in promoting algal growth and ultimately eutrophication of waterbodies. While the classical example here is from the 1960s–1970s, where the U.S. detergent industry used various strategies to manufacture uncertainty around the links between phosphates in detergents and eutrophication of lakes (Eberly 1974), this issue also became a source of delay for mitigating Baltic Sea eutrophication. Saunders et al. (2017) report a scientist asserting that detergent manufacturers made attempts in the 1970s to delay scientific consensus on Baltic Sea eutrophication by commissioning their own, often poorly designed, research which claimed that phosphorus does not promote algal growth. Elements of substantial science critique and what can be classified as implicatory denial can also be observed in the ongoing debates on how international agreements on nutrient reductions can be implemented in specific sectors like agriculture (Saunders et al. 2017).

The patterns of science denial as a delay mechanism in environmental governance thus seem quite similar for a number of issues, but research on other issues than climate denial is sparse and little is known about effective counteracting strategies (Edvardsson et al. 2017). It seems well-motivated to further explore the delay mechanism of science denial in environmental governance research and practice.

### Decision thresholds

In environmental policy, it is commonplace to require a vast amount of scientific evidence before measures are decided. Even in cases when democratically agreed goals are not achieved, the burden of proof for showing that proposed decisions yield socioeconomic improvements commonly rests with those suggesting further measures, rather than with those advocating business as usual. While it is important to base policies on science as far as possible, high requirements on knowledge and data may, particularly in cases of scientific uncertainty, seriously delay policies that promote environmental goal achievement. To cope with such decision situations, the precautionary principle has been held forward since long, also in legislation, but it is far from always applied (Sandin et al. 2002; Karlsson 2005; Alfredsson and Karlsson 2016; Stirling and Coburn 2018). Three key questions in this context concern how much evidence that is needed before decision-making, where the burden of proof is to be placed, and which institutions or groups that are charged with making decisions.

In chemical policy, the conventional risk paradigm (Karlsson and Gilek 2018) is built around risk assessments, which are dependent on large amounts of data. Comprehensive risk assessments are in turn a common requirement for risk management decisions, which in public policy regularly are associated with a strong burden of proof being placed on policy-makers and public agencies. Studies show that such decision thresholds are very difficult to pass, due to a striking lack of available data on substance properties, in particular concerning combination effects (Gilbert 2011; Kortenkamp and Faust 2018). This is obvious in the European Union as well as in the U.S., with resulting delay effects in chemicals policy and in relation to public goals (Karlsson 2010; Woutersen et al. 2018). In order to cope better with a situation like this, full implementation of precautionary strategies has been proposed (Karlsson 2010; Stirling and Coburn 2018).

A similar situation exists in climate policy, where instead cost–benefit analyses (CBA) are commonly required before decisions are made (Alfredsson and Karlsson 2016; van den Bergh 2017). To start with, the exact costs and benefits of climate policy decisions are often very difficult, if not impossible, to determine, due to the striking scientific uncertainty at hand, in particular when it comes to assessing impacts and costs of large changes in average global temperatures and Earth systems (Schellnhuber et al. 2016). Moreover, some key economic aspects are sparsely studied, for example the co-benefits that have been shown to commonly follow with climate policies, resulting in data shortages on relevant parameters in CBA-based decision-making (Karlsson et al. unpublished). When the actual burden of proof additionally rests with any stakeholder or public agency proposing, for example, additional climate policy instruments, such as carbon taxation in various sectors, CBA-requirements are likely to function as decision thresholds, causing serious delays in goal achievement as well as a biased decision-making context (Alfredsson and Karlsson 2016).

To prevent delay and promote goal achievement, science-based precautionary strategies can be applied, which mean that asymmetric data requirements are not accepted as valid for decision-making unless the burden of proof is placed on those opposing further policies and measures, e.g. in cases of huge threats and scientific uncertainty (Karlsson 2005, 2010; Alfredsson and Karlsson 2016; van den Bergh 2017; Stirling and Coburn 2018). This insight on decision thresholds as delay mechanisms is valid for other complex environmental decision-making situations than climate and chemical policy, for example, when it comes to policies that aim to mitigate eutrophication and manage water bodies and forests (e.g. Keskitalo and Pettersson 2012; Saunders et al. 2017; Klapwijka et al. 2018). Further studies of decision-threshold delay could thus prove valuable for research and practice in environmental governance.

## Discussion

This Perspectives article is largely based on studies that we and others have done on goal-state gaps in environmental governance, and on why delay is occurring and is difficult to cope with. The new framework that we present can hopefully be used in further governance research, for structuring the analysis of lack of goal achievement in additional case studies and more in-depth than we have done in our brief illustrations of four wicked environmental problems linked to the two delay mechanisms of science denial and decision thresholds.

As we have argued and exemplified throughout this text, focusing on delay mechanisms holds several analytical merits and complement previous research concepts and approaches that explore gaps, e.g., governance barriers, and scientific uncertainty and disagreement. Importantly though, our argument is not that these various goal-impeding factors are not useful to study. We rather contend that our framework allows additional comprehensive and integrated analyses of goal-failures across a board of key interacting processes in science and policy, and as a means to structure comparisons of different issue areas in environmental governance. First, the framework opens for analysis of both individual delay mechanisms and the overall outcome within a governance system. Thereby, new avenues for coping with delay may be identified and elaborated. Second, the framework illustrates that delay mechanisms exist in both the science and the policy domain, which calls for inter- and transdisciplinary research. This may help overcome a still frequent shortcoming in much previous environmental governance-related research, where natural sciences usually focus on e.g., assessing the state of the environment and social sciences more commonly aim at e.g., understanding socio-political barriers, often without these research fields having much contact with each other.

However, we also want to underline that the framework is indeed new, and as such in need of development, refinement and operationalisation. Besides the delay mechanisms, we have described briefly, and exemplified more in depth in two cases, other parameters are certainly relevant to include, as are the interactions between them. It is similarly important to give attention to the surrounding environment of stakeholders, which evidently includes actors with different roles and agendas. Some of these, for example, operate to organise science denial actively (Edvardsson et al. 2017; Karlsson 2019), whereas other strive within agencies, ministries and public inquiries to cope with decision thresholds (Karlsson and Westling 2017; Karlsson et al. unpublished). There is thus a need to identify and highlight the roles of different stakeholder groups, for example, in relation to science denial and decision thresholds.

While there is a consequent need for further academic refinement of the proposed framework, we believe that it already can form a useful basis for policy review and development. The succinct design of the framework makes it easy to apply for policy-makers in for e.g., agencies, inquiries, ministries and parliaments. For them, delay mechanisms are often practically relevant since they both cause goal failures and highlight governance challenges. The urgency of the need for more systematic and routine analysis of delay in environmental policy is obvious, considering how large and common goal-state gaps often are, from national to international levels. In the case of Sweden, even though gap analysis to some extent already is part of the procedures of the Swedish Environmental Objectives System, more remains to be done in that respect, bearing in mind that all but one of the objectives repeatedly agreed by the parliament are considered to be out of reach (SEPA 2019). The challenges elsewhere are often much larger. It would, therefore, be valuable if instructions for policy review and development in different governance contexts can be complemented with requirements to analyse potential delay mechanisms behind goal failures, from local policy evaluations to impact assessments within the European Union or the UN system. In doing so, overlooked causes behind goal-state gaps may be identified, potentially promoting gap closure. Evidently, any application of the framework needs to be contextually informed. A key message is nevertheless that delay mechanisms need to be put more in the spotlight in environmental governance, to not only mind but also better bridge the common environmental goal-state gaps.

## Footnotes

1 Outside the area of environmental governance, science denial has been common in relation to e.g. risks of tobacco consumption (Edvardsson et al. 2017).

2 A researchers’ roundtable on the theme “Closing the science-policy gap for endocrine disrupting chemicals: denial and delay”, arranged in Stockholm on March 14, 2017, provided several stories on science denial, linked to e.g. brominated flame retardants and bisphenols.
